# LA-EAD: Simple and Effective Methods for Improving Logical Anomaly Detection Capability

**DOI:** 10.3390/s25165016

**Published:** 2025-08-13

**Authors:** Zhixing Li, Zan Yang, Lijie Zhang, Lie Yang, Jiansheng Liu

**Affiliations:** 1 School of Advanced Manufacturing, Nanchang University, Nanchang 330031, China; lizhixing@email.ncu.edu.cn (Z.L.);; 2 Tellhow Sci-Tech Co., Ltd., Nanchang 330096, China

**Keywords:** anomaly detection, logical anomaly, knowledge distillation, deep learning

## Abstract

In the field of intelligent manufacturing, image anomaly detection plays a pivotal role in automated product quality inspection. Most existing anomaly detection methods are adept at capturing local features of images, achieving high detection accuracy for structural anomalies such as cracks and scratches. However, logical anomalies typically appear normal within local regions of an image and are difficult to represent well by the anomaly score map, requiring the model to possess the capability to extract global context features. To address this challenge while balancing the detection of both structural and logical anomalies, this paper proposes a lightweight anomaly detection framework built upon EfficientAD. This framework integrates the reconstruction difference constraint (RDC) and a logical anomaly detection module. Specifically, the original EfficientAD relies on the coarse-grained reconstruction difference between the student and the autoencoder to detect logical anomalies; but, false detection may be caused by the local fine-grained reconstruction difference between the two models. RDC can promote the consistency of the fine-grained reconstruction between the student and the autoencoder, thereby effectively alleviating this problem. Furthermore, in order to detect anomalies that are difficult to represent by feature maps more effectively, the proposed logical anomaly detection module extracts and aggregates the context features of the image, and combines the feature-based method to calculate the overall anomaly score. Extensive experiments demonstrate our method’s significant improvement in logical anomaly detection, achieving 94.2 AU-ROC on MVTec LOCO, while maintaining strong structural anomaly detection performance at 98.4 AU-ROC on MVTec AD. Compared to the baseline, like EfficientAD, our framework achieves a state-of-the-art balance between both anomaly types.

## 1. Introduction

Image anomaly detection, as an important task in the field of machine vision, has the core objective of identifying anomalous regions within an image or providing a prediction score on the existence of anomalies in an image. This technique has demonstrated excellent application value in numerous scenarios [1,2,3]. Especially in industrial anomaly detection scenarios, there are diverse forms of product defects, typically including cracks, dirt, scratches, and so forth. Currently, deploying anomaly detection algorithms into intelligent vision sensors to enable online inspection of anomalies on production lines has become a major trend. Consequently, both accuracy and latency are critical considerations.

The defect detection technology represented by supervised methods such as object detection and instance segmentation has achieved significant progress in detection accuracy and efficiency [4,5,6,7]. However, in real industrial environments, it is more common for defect samples to be scarce and for anomaly samples to be expensive to collect, so that for a long period of time, models can only be trained using artificial samples. This situation greatly restricts the effective use of supervised learning in production. Supervised learning demands extensive sample labeling, which is another crucial factor contributing to its high cost. Therefore, unsupervised anomaly detection methods have become a popular research direction in the field of industrial vision [8,9], which rely only on normal samples for model training and then make predictions in the absence of a priori knowledge of anomalous features.

Image anomalies can be classified into structural and logical anomalies [10]. Structural anomalies are usually characterized by changes in the texture or shape of the product, with typical defects such as cracks. This has long been a focus of attention for many researchers, and the release of multiple benchmarks such as MVTec AD and KolektorSDD [11,12,13,14] has led to rapid development in structural anomaly detection methods. In contrast, logical anomalies have merely begun to attract the attention of researchers quite recently. Logical anomalies typically denote the abnormal positions or disordered arrangement of normal components, with typical defects such as the mislabeling of different products [15]. Most of the current anomaly detection methods are focused on extracting local features to enhance the ability to detect structural anomalies, while detecting logical anomalies requires the model to have superior global information extraction capability to comprehensively capture the logical relationships among multiple objects [16].

The anomaly detection methods for the above anomaly types mainly include reconstruction-based [17,18,19] and feature-based [20,21,22] methods. In reconstruction-based methods, the training phase uses normal images to train the image reconstruction model. When anomalous images are input, it is difficult for the model to correctly reconstruct the anomalous area. Thus, in the predicting phase, by comparing the differences between the reconstructed image and the input image, the probability of defect existence can be estimated, and the segmentation of the defect area can be achieved by setting a different threshold. Feature-based methods utilize deep neural networks to extract high-dimensional features to improve tolerance for noise, and construct classification surfaces or use feature distances to predict anomalies. Based on these methods, many anomaly detection models have been proposed. However, most of these methods cannot achieve a good balance in both structural anomaly and logical anomaly detection, and usually only perform well in detecting one type of them. Furthermore, the inference latency of the model is also an important factor for its implementation in production scenarios. In this paper, the proposed method combines reconstruction-based and feature-based methods.

Achieving a balance between structural anomaly detection and logical anomaly detection constitutes a challenging task. Bergmann et al. [10] designed GCAD based on knowledge distillation by establishing a teacher–student architecture composed of local and global branches to address both types of anomaly detection requirements. Building on the GCAD method, Batzner et al. [23] further proposed EfficientAD, in which they designed a lightweight and efficient feature extraction network, PDN, while introducing hard feature loss and penalty constraints to effectively improve the detection performance of the teacher–student architecture. Meanwhile, owing to the high efficiency of PDN networks, the low latency and high throughput of EfficientAD make it the preferred solution for practical applications. Although EfficientAD has emerged as a new SOTA method, experimental results on the MVTec LOCO dataset show that there is still considerable room for improvement in the logical anomaly detection capability.

**Figure 1:**
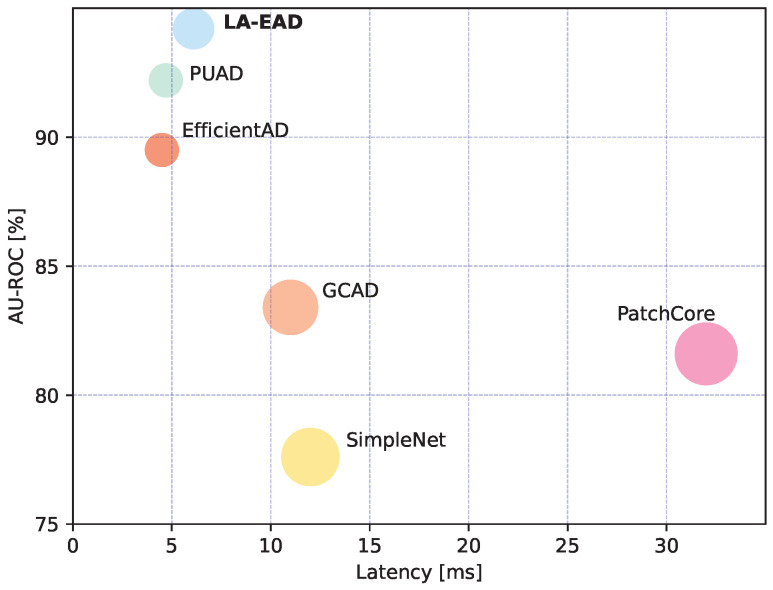
AU-ROC performance vs. latency on the MVTec LOCO dataset. The size of the circles correspond to the number of model parameters. The latency is measured on an NVIDIA RTX A6000 GPU with an input image size of 256 × 256. Our method achieves the best latency/accuracy trade-off, which undoubtedly facilitates the deployment and application of this algorithm in industrial production environments.

Based on the above analysis, in view of the high detection accuracy and low latency of EfficientAD, we select EfficientAD as our baseline and propose our improved method, LA-EAD (improved EfficientAD for logical anomalies). Figure 1 presents the performance of LA-EAD and various SOTA methods in terms of accuracy and efficiency for logical anomaly detection. Due to the characteristics of the autoencoder structure, there is a systematic reconstruction difference between the autoencoder’s output and the teacher’s output, namely, the autoencoder tends to reconstruct coarsely. The student network possesses a smaller receptive field, enabling it to extract local features in a more refined manner. Based on the above characteristics, EfficientAD detects logical anomalies by the difference between the output of the autoencoder and the student network. However, due to the smaller receptive field of the student network and the information flow difference caused by knowledge distillation, the student may not learn the systematic reconstruction difference between the autoencoder and the teacher, i.e., the student may reconstruct the image too finely. The difference map between the student and the autoencoder is prone to false-positive results. In order to alleviate this problem, we propose the RDC, which prompts the student to acquire the coarse-grained reconstruction features of the autoencoder, thereby mitigating the false-positive phenomenon.

**Figure 2:**
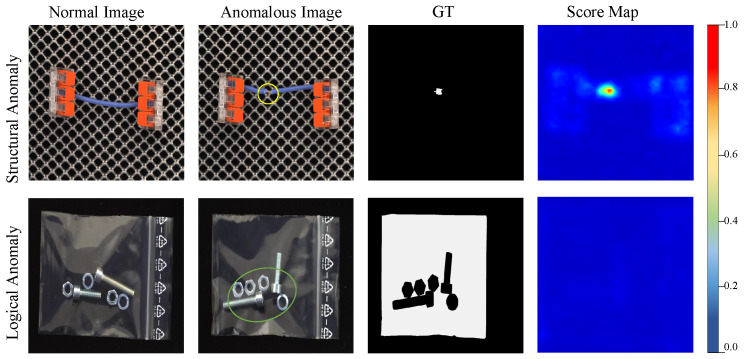
Representative samples of structural anomalies and logical anomalies are selected from MVTec LOCO, and score maps are generated by EfficientAD. The breakage of the connecting line can be clearly visualized in the spatial feature map. However, incorrect numbers of nuts and gaskets cannot be clearly marked. As shown in the label map, even humans are unable to clearly mark the location where the anomaly occurs on the map.

On the other hand, most logical anomalies are difficult to intuitively represent by spatial feature maps. Sugawara et al. [24] defined these as unpicturable anomalies. Figure 2 shows the anomaly score maps generated by EfficientAD for structural anomalies and logical anomalies, respectively. We observe that structural anomalies can be intuitively represented by spatial feature maps. However, for logical anomalies, spatial feature maps are unable to accurately describe the locations where the anomalies occur. For anomalies such as the incorrect number of thumbtacks and the incorrect combination of parts, even humans cannot determine how the abnormal features should be labeled on the feature map. Intuitively, logical anomalies are inherently difficult to describe by anomaly score maps. For such anomalies, reconstruction-based methods represented by EfficientAD are unable to achieve a high accuracy rate. Based on the above analysis, we propose the logical anomaly detection module, which contains a global–local feature extractor and integrates the feature-based detection method into EfficientAD.

Our contributions are summarized as follows:

1. We propose a reconstruction difference constraint to alleviate the false detection caused by the background reconstruction difference between the student and the autoencoder, which can be seamlessly integrated into the training phase of EfficientAD.
2. We propose a detection module for logical anomalies, which contains a modeling–evaluating process. For the modeling process, we design an efficient feature extractor that can effectively extract context information. The logical anomaly detection module is integrated into the designed LA-EAD anomaly detection framework.
3. Our extensive experimental results demonstrate that LA-EAD significantly improves the accuracy for detecting logical anomalies while maintaining a high accuracy for detecting structural anomalies, making it highly competitive in terms of efficiency and performance.

## 2. Related Works

### 2.1. Reconstruction-Based Anomaly Detection Methods

The main idea behind reconstruction-based methods is to train the model using normal samples and make it difficult for the model to reconstruct the unlearned anomaly features when presented with anomaly input, thus producing a difference map in the abnormal region. The models used for reconstruction are usually generative models represented by GAN and an autoencoder, and a number of studies have been conducted based on them [25,26,27,28]. Zavrtanik et al. [29] considered anomaly detection as an integrated issue of reconstruction and discrimination, and proposed DRAEM, which learns the reconstruction of normal images and decision bounds for anomalous samples. In order to accomplish a more realistic reconstruction, Zavrtanik et al. [30] further proposed a dual-decoder based architecture, DSR, which was designed to process two different appearance subspaces by two decoders in parallel to reconstruct normal images with high fidelity. To avoid false-positive results due to inaccurate reconstruction, Liu et al. [31] proposed SimpleNet to synthesize anomalies with the assistance of pre-trained features to strengthen the judgment of the discriminator model. The above methods focus on structural anomaly detection while ignoring logical anomalies. GCAD and EfficientAD train the teacher–student networks based on knowledge distillation and utilize the difference map between the teacher and the student to achieve two types of anomaly detection. EfficientAD designs a simple and efficient teacher–student network for detecting structural anomalies and introduces an autoencoder for the purpose of detecting logical anomalies.

The aforementioned reconstruction-based methods can effectively handle structural anomalies, yet they exhibit the following limitations in logical anomaly detection. The optimization objective of reconstruction models focuses on pixel-level or local structural appearance consistency, and their reconstruction errors primarily reflect whether the elements themselves are abnormal. In contrast, the essence of logical anomalies lies in the violation of global logical relationships, where the elements themselves may be completely normal. Consequently, the reconstruction errors are often insignificant, making it difficult for the models to capture such anomalies.

Although EfficientAD attempts to address logical anomalies, its mechanism for detecting such anomalies via an autoencoder still relies on the assumption that normal logical features can be reconstructed. Nevertheless, the training of the autoencoder still centers on the consistency of appearance feature distributions and lacks modeling of implicit logical constraints, resulting in logical anomaly detection performance still needing improvement.

### 2.2. Feature-Based Anomaly Detection Methods

The feature-based method extracts the features of normal samples in the feature space as templates and measures the distance [32,33] or constructs the classification surface [34,35] for the normal and anomalous samples. Cohen et al. [36] put forward a novel anomaly detection method named SPADE, which uses a pre-trained model to extract multi-scale features to construct a memory bank and calculates the anomaly score in the local area through nearest neighbor search. Its advantage is that multi-scale feature matching can improve the positioning accuracy, while the disadvantages are high memory consumption and insufficient modeling of the global context. To mitigate the computational overhead, PaDiM [20] establishes a multivariate Gaussian distribution model of image blocks and quantifies the anomaly score by using the Mahalanobis distance, which greatly reduces the time consumption of the algorithm. Based on PaDiM, PatchCore [22], which has a higher accuracy, was proposed. For background modeling, PatchCore applies coreset downsampling to extract the most representative feature vectors, which further reduces the computational complexity and memory utilization. The above-mentioned feature-based method relies on the statistical distribution of local features. Its anomaly score only reflects whether the local features deviate from the normal distribution. Therefore, it has achieved good results in structural anomaly detection, but lacks optimization for logical anomalies.

To deal with logical anomalies, some feature-based logical anomaly detection methods have been proposed. SINBAD [37] regards logical anomalies as anomalies composed of normal elements. From this perspective, SINBAD uses a pre-trained network to extract the features of the samples to be tested and model them as an unordered set of elements described by a histogram, with the anomaly score defined as the Mahalanobis distance between the normal and anomalous samples. The test results of SINBAD on the MVTec LOCO shows complementary advantages to the reconstruction-based approach. However, SINBAD requires the design of complex histogram modeling strategies and is difficult to integrate into other frameworks. Further, Sugawara et al. [24] proposed PUAD, which combines EfficientAD with a feature-based approach. For unpicturable anomalies, PUAD uses the former half of the student network in the EfficientAD framework to extract features, models normal samples as templates, and calculates the Mahalanobis distance between the test sample and the template as the anomaly score. PUAD improves the logical anomaly detection performance of EfficientAD, but it fails to optimize the global feature extraction capability of EfficientAD’s existing network structure.

To improve the capability of logical anomaly detection, we analyze the deficiencies of existing methods and propose an improvement method from the perspective of combining reconstruction-based and feature-based methods. Our method does not require a label for training and can be simply incorporated into existing works.

## 3. Method

**Figure 3:**
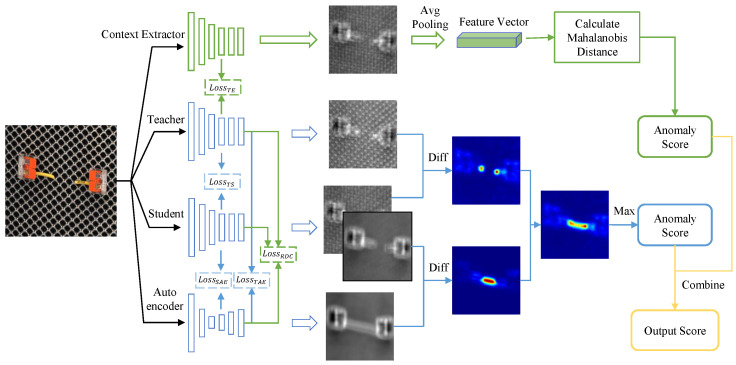
Overall framework of LA-EAD. LA-EAD introduces an additional context feature extractor based on the architecture of EAD. During the training process, the RDC is applied while the feature extractor is trained by knowledge distillation with the teacher model. During the prediction process, the score of the EfficientAD and the score of the logical anomaly detection module are combined to output the final score. The major contributions presented in this paper are marked in green.

The proposed LA-EAD framework consists of all the components of EfficientAD and an additional global context feature extractor, as shown in Figure 3. The structure and training methods belonging to the EfficientAD section refer to the original paper [23]. During the training stage, the weights of the teacher model are frozen, and normal images are input into the teacher, student, autoencoder, and context feature extractor, respectively. Following the training method of EfficientAD, the teacher model uses normal samples to guide the student model to learn local normal patterns through knowledge distillation, while the autoencoder learns the global normal patterns from the student model. Furthermore, under the guidance of the teacher model, the feature extractor acquires the ability to extract context features through knowledge distillation, and then establishes the global feature representation of all normal samples as the template. During the testing stage, following the anomaly map calculation method of EfficientAD, the local anomaly map and global anomaly map are calculated, respectively, and integrated into the final anomaly map. The proposed feature extractor captures and integrates global semantic features from input images, while computing their distance to normal samples’ global feature templates as the anomaly score for logical anomaly detection. The final anomaly score is obtained by fusing this logical detection output with EfficientAD’s anomaly prediction score through weighted summation.

### 3.1. Lightweight Teacher–Student Network

EfficientAD designs an anomaly detection architecture based on knowledge distillation, which includes a teacher–student network and an autoencoder. It leverages the information variability introduced by knowledge distillation for anomaly detection. Both the teacher and student models adopt the PDN structure, an efficient feature-extraction convolutional network designed for EfficientAD. As shown in Figure 3, the teacher model can reconstruct input images at a fine-grained level. The student model is divided into two parts, the former portion (teacher branch) and the latter portion (AE branch). Since models are trained only on normal images, the former portion of the student model learns from the teacher model the fine-grained reconstruction capability of normal images, and the difference map between them is used to detect fine-grained structural anomalies. Meanwhile, the autoencoder learns the global reconstruction capability of normal images from the teacher model, and the latter portion of the student model learns the global reconstruction capability of normal images from the autoencoder. Since the receptive field of the student model is smaller, the student model demonstrates superior capability in reconstructing local features compared to the autoencoder, and it is the reconstruction difference between the two that is exploited by EfficientAD for the detection of logical anomalies.

### 3.2. Reconstruction Difference Constraint

The autoencoder has difficulty reconstructing fine-grained features, and using the difference map between the teacher and autoencoder as the score map for logical anomalies will trigger more erroneous results. Therefore, EfficientAD adds an output branch to the student model, where the student learns the reconstructed features of the autoencoder on normal samples and reduces false-positive results.

**Figure 4:**
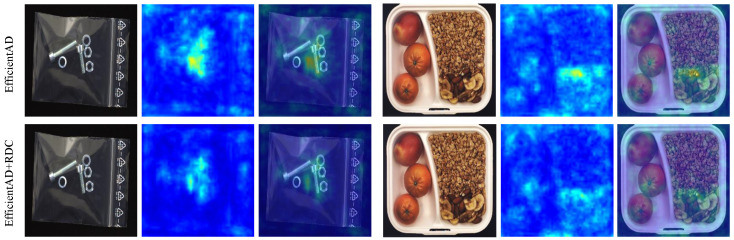
Comparison of anomaly score maps on normal images before and after introducing the RDC. We use the original EfficientAD and EfficientAD with the introduction of RDC to perform inference on the same normal image and compare the score maps, respectively. The results show that, as we expected, after the introduction of RDC, the score responses on the normal images are significantly decreased, and the problems of false positives are effectively alleviated.

In summary, based on the knowledge distillation strategy, the autoencoder learns to reconstruct images from the teacher model, while the student learns reconstruction capabilities from the autoencoder. There is a systematic reconstruction difference between the autoencoder and the teacher, which is expected and utilized by EfficientAD. However, due to the structure of the student network, it focuses on the local fine-grained features of the image, and there is inevitably a difference in information flow between the autoencoder and the student. Therefore, the student finds is difficult to learn the systematic reconstruction difference of the autoencoder relative to the teacher model. Figure 4 shows that when testing with normal samples, there are areas with high response in the anomaly prediction map of EfficientAD. The predicted results of these areas may be considered detected anomaly features, but in reality, they belong to false-positive results.

We want the student’s AE branch to learn as much as possible about the systematic reconstruction bias of the autoencoder on normal images, thus reducing false-positive results. For this purpose, this paper proposes the reconstruction difference constraint acting between the student and the autoencoder, which is computed as follows. First, given the normal image *I*, the difference $D_{TAE}$ between the teacher’s output and the autoencoder’s output is computed:

(1) $$D_{TAE}={\left(T\left(I\right)-A\left(I\right)\right)}^{2},$$

where T(I) and A(I) denote the output features of the teacher model and the autoencoder for normal sample images, respectively, with their number of channels, height, and width represented by *C*, *H*, and *W*. Then, let $S_{AE}$ denote the student’s AE branch; the difference $D_{TSAE}$ between the student’s output and the teacher’s output is calculated as follows:

(2) $$D_{TSAE}={\left(T\left(I\right)-S_{AE}\left(I\right)\right)}^{2}.$$

Finally, the RDC loss $L_{RDC}$ can be calculated using the MSE loss. The calculation formula is as follows:

(3) $$L_{RDC}=\frac{1}{CHW}\sum_{c}{\left(D_{TAE}-D_{TSAE}\right)}^{2}.$$

We fuse the defined reconstruction difference constraint into the original loss function of EfficientAD, and this constraint guides both the student and the autoencoder to learn to approximate the reconstructed difference from the teacher. The student and the autoencoder obtain the score maps used for logical anomaly detection by making a difference operation. The reconstruction difference constraint will motivate the student and autoencoder to offset the reconstruction difference when making a difference, thus effectively mitigating the false-positive problem that exists in the original EfficientAD in detecting logical anomalies.

### 3.3. Logical Anomaly Detection Module

For the unpicturable anomalies mentioned above, we design a logical anomaly detection module and integrate it into the teacher–student network described in Section 3.1. It is worth mentioning that with a simple adaptation, our logical anomaly detection module can be integrated into numerous anomaly detection architectures.

#### 3.3.1. Modeling–Evaluating Process

Inspired by Cohen’s works [37], suggesting that feature-based and reconstruction-based approaches may have complementary advantages in detecting logical anomalies, we introduce a feature-based approach and design our logical anomaly detection module. Characterizing the degree of deviation between normal and abnormal distributions using the Mahalanobis distance is an effective method in the field of anomaly detection. Such methods usually use the output features of a pre-trained deep neural network classifier to represent the sample distribution, assuming that the sample distribution follows a Gaussian distribution, and defining the Mahalanobis distance between the sample to be tested and the normal sample distribution as the anomaly score.

The modeling–evaluating process of our logical anomaly detection method is as follows. First, we extract a feature map using the designed feature extractor over all the samples on the set of normal samples and apply global average pooling to the feature map to aggregate the features to obtain a global feature representation corresponding to each sample.

Let $f_{c,w,h}\in R^{C\times W\times H}$ denote the proposed feature extractor’s output, where *C*, *H*, and *W* are the output’s channel, height, and width, respectively.

(4) $$f_{n}={\left(HW\right)}^{-1}\sum_{h=1}^{H}\sum_{w=1}^{W}f_{c,w,h}.$$

We consider the normal sample set with *N* samples as the template. Then, all the feature vectors in the set are averaged as the mean vector $f_{m}$ of the template’s feature vectors:

(5) $$f_{m}=\frac{1}{N}\sum_{n=1}^{N}f_{n}.$$

Then, all features are centralized in the vector set by subtracting the mean vector from each feature vector to obtain centralized feature vectors:

(6) $${\tilde{f}}_{n}=f_{n}-f_{m},$$

where ${\tilde{f}}_{n}$ denotes the centralized feature vector. Then, the covariance matrix Σ of the feature vectors set is computed as follows, where $\tilde{X}$ denotes the matrix consisting of all centralized feature vectors:

(7) $$\Sigma =\frac{1}{N-1}{\tilde{X}}^{T}\tilde{X}.$$

In the process of anomaly detection, the proposed feature extractor is used to extract the feature map of the test sample, and the global average pooling is used to obtain the feature vector $f_{test}$, as shown in Equation (4). Both the feature vector $f_{test}$ of the test samples and the template’s mean vector $f_{m}$ are employed to calculate the Mahalanobis distance as the anomaly score:

(8) $$M\left(f_{test}\right)=\sqrt{{\left(f_{test}-f_{m}\right)}^{T}\Sigma^{-1}\left(f_{test}-f_{m}\right)}.$$

#### 3.3.2. Multi-Scale Outlook Attention

**Figure 5:**
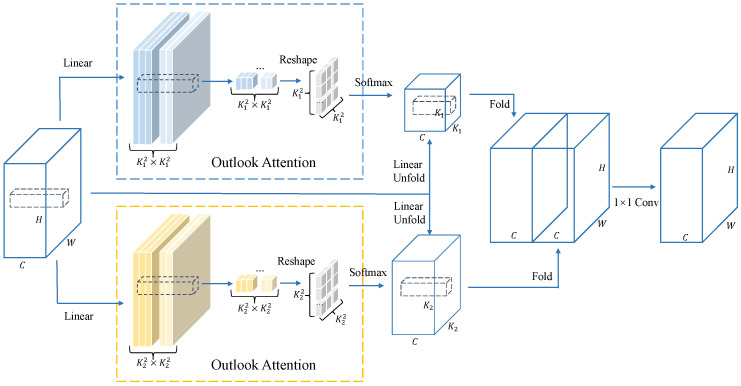
Illustration of the MSOA module.

Unlike structural anomalies, images containing logical anomalies usually exhibit normal features in a local region but behave abnormally when viewed globally. Based on this property, our human eyes naturally perceive logical anomalies by global observation. Therefore, we believe that detecting logical anomalies requires models with better global perception. From this intuition, we design a multi-scale outlook attention module (MSOA module) that is capable of effectively extracting fine-grained global contextual features. The structure of the feature extractor is shown in Figure 5. And we embed it into a feature extraction network, which is used to extract global–local features and applied to the logical anomaly detection method described in Section 3.3.1.

Li et al. [38] proposed a novel attention mechanism for fine-grained feature learning, outlook attention, which is simple and lightweight, and can effectively encode fine-grained information while aggregating contextual information. Outlook attention has excellent performance in image classification and segmentation tasks, with fine-grained feature extraction and contextual information aggregation capabilities. However, the feature extraction window of the original outlook attention is a fixed single scale. In order to further improve the contextual information aggregation ability of the model, we use two feature extraction windows of different scales to synchronously extract features, and after processing the features through two outlook attention blocks, we obtain two enhanced feature maps of exactly the same size. Finally, we use a 1 × 1 convolutional layer to fuse the features as the enhanced feature map output of the MSOA module.

#### 3.3.3. Feature Extractor

**Figure 6:**
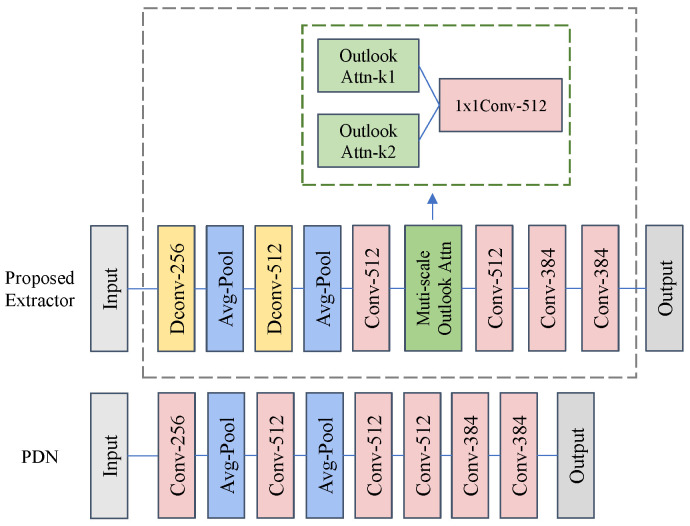
Comparison of the structure between our proposed feature extractor and PDN. We introduce dilated convolution to increase the sense field and aggregate contextual features. Meanwhile, average pooling is performed at the shallow layer of the network to reduce the feature map size and speed up the training and inference. Embedding our designed multi-scale outlook attention mechanism in the deeper layer of the network can further integrate the global context features.

The PDN network proposed in EfficientAD is a CNN architecture composed of convolutional layers and pooling layers. PDN has the characteristics of high efficiency and lightness, and its inference efficiency is the key reason why EfficientAD can achieve low latency. For logical anomalies, PUAD [24] simply utilizes the former half of the student network (PDN) to extract features for calculating the Mahalanobis distance. However, detecting logical anomalies requires the model to utilize a large receptive field to obtain global context information, while also obtaining fine-grained features as much as possible. PDN network has insufficient capability in these areas. We hope to maintain the efficiency of the feature extraction model while enhancing its capability as previously mentioned. Therefore, based on the main architecture of PDN, we design a feature extractor suitable for logical anomaly detection, which is shown in Figure 6.

Similar to the student model, we direct the feature extractor to learn knowledge from the teacher model by minimizing the difference between the outputs of the feature extractor and the teacher model, as shown in Figure 3. In this process, the MSOA module prompts the network to pay more attention to global context features. The reconstruction loss function $L_{TE}$ between the feature extractor and the teacher is shown below, where E(I) denotes the output feature of the proposed feature extractor:

(9) $$L_{TE}={\left(CHW\right)}^{-1}\sum_{c}{∥T{\left(I\right)}_{c}-E{\left(I\right)}_{c}∥}^{2}.$$

In industrial production environments, new product categories are usually added from time to time, which requires field personnel to retrain the model for the new product categories. The penalty loss defined in EfficientAD requires loading the large dataset ImageNet [39], which is extremely inconvenient for deployment and training across different devices. Considering the convenience of algorithm deployment and to accelerate training convergence, we remove the penalty loss. If users deploy and train our model on different devices again, they only need to copy the pre-trained teacher model.

Finally, the overall loss function for a given training sample is defined as follows, where α is a pre-determined constant coefficient. Following the loss function design of EfficientAD, in this study, $L_{TS}$ represents the reconstruction loss between the student model and teacher model, $L_{TAE}$ represents the reconstruction loss between the autoencoder and teacher model, and $L_{SAE}$ represents the reconstruction loss between the student model and autoencoder.

(10) $$L_{\mathrm{total}}=L_{TS}+L_{TAE}+L_{SAE}+L_{TE}+\alpha L_{RDC}.$$

In the inference phase, the core process of LA-EAD is shown in Algorithm 1. Firstly, the proposed feature extractor *E* is used to extract features from the input image *I*, obtaining the feature $f_{c,w,h}$. Then, the global average pooling operation (as shown in Equation (4)) is applied to compute the feature vector $f_{test}$. Secondly, based on the precomputed feature vector $f_{m}$ and covariance matrix Σ, the anomaly score $s_{u}$ of the logical anomaly detection module is calculated using the Mahalanobis distance (as shown in Equation (8)). Next, the image *I* is fed into the EfficientAD model to obtain the output anomaly score $s_{p}$. Finally, referring to the method in PUAD, $s_{p}$ and $s_{u}$ are normalized separately using the mean and variance of the validation set, and the normalized scores are summed to get the final anomaly score $s_{\mathrm{final}}$. This process achieves a comprehensive judgment of image anomalies by fusing the outputs of the logical anomaly detection module and EfficientAD.
**Algorithm 1** LA-EAD inference process**Input:** input image *I*, EfficientAD model EAD, proposed extractor *E*, precomputed covariance matrix Σ, precomputed feature vector $f_{m}$, precomputed normalization parameters $\mu_{p}$, $\sigma_{p}$, $\mu_{u}$ and $\sigma_{u}$
**Output:** final anomaly score $s_{\mathrm{final}}$
  1:**Extract features from the proposed extractor *E***  2:Get output feature of the proposed extractor for *I*:  3:    $f_{c,w,h}=E\left(I\right)$  4:Compute global average pooled feature vector:  5:    $f_{test}={\left(HW\right)}^{-1}\sum_{h=1}^{H}\sum_{w=1}^{W}f_{c,w,h}\;(\mathrm{as}\;\mathrm{Equation}\;\left(4)\right)$  6:**Calculate anomaly score $s_{u}$ of the logical anomaly detection module:**  7:Compute Mahalanobis distance using precomputed $f_{m}$ and $f_{test}$:  8:    $s_{u}=\sqrt{{\left(f_{test}-f_{m}\right)}^{T}\Sigma^{-1}\left(f_{test}-f_{m}\right)}\;(\mathrm{as}\;\mathrm{Equation}\;\left(8)\right)$  9:**Get anomaly score $s_{p}$ from EfficientAD**10:Feed the image *I* into EfficientAD for the inference process and obtain the anomaly score:11:    $s_{p}=EAD\left(I\right)$12:**Normalize and fuse scores**13:Perform separate normalization on the $s_{p}$ and $s_{u}$, with the normalization parameters (mean and variance) calculated from the inference results of the validation subset. (following PUAD [24])14:    $s_{p}^{\mathrm{norm}}=\frac{s_{p}-\mu_{p}}{\sigma_{p}},\;s_{u}^{\mathrm{norm}}=\frac{s_{u}-\mu_{u}}{\sigma_{u}}$15:Compute final anomaly score:16:    $s_{\mathrm{final}}=s_{p}^{\mathrm{norm}}+s_{u}^{\mathrm{norm}}$17:**return** $s_{\mathrm{final}}$

## 4. Experimental Result Analysis and Discussion

### 4.1. Datasets and Metrics

We evaluate our proposed method on two popular public datasets, MVTec LOCO and MVTec AD. MVTec LOCO contains five product categories, each consisting of structural anomalies and logical anomalies. Since this dataset contains a diversity of logical anomaly samples, it is currently the dominant dataset for evaluating the logical anomaly detection capability of algorithms. The entire dataset consists of 1772 normal images for training, 304 normal images for validation, and 432 structural anomaly images and 561 logical anomaly images for evaluation, respectively.

MVTec AD contains multiple anomaly scenarios for 15 product categories with a total of 3629 normal images for training and 1725 anomaly images for testing. The test set contains multiple categories of anomalies in the form of holes, scratches, and marks, totaling 70 types of anomalies.

We use the image-level AU-ROC (area under the receiver operator curve) metric to evaluate the binary classification ability of all compared algorithms with respect to the presence or absence of anomalies in an image.

### 4.2. Implementation Details

Since the main framework of LA-EAD is derived from EfficientAD, we follow EfficientAD’s settings for the training hyperparameters. EfficientAD-M refers to the medium-scale version of EfficientAD, which exhibits better detection performance than EfficientAD-S. Therefore, the default architecture of LA-EAD is built based on EfficientAD-M. In the image preprocessing stage, we resize the training image to 256 × 256 pixels, normalize it using the known mean and standard deviation of the ImageNet dataset, and also perform sample enhancement of the input image in terms of brightness, contrast, and saturation. In the training phase, we set the batch size to 1 and the number of output channels to 384. We train our models using 70000 iterations on a single A6000 GPU, and we adopt the Adam optimizer and set the learning rate and weight decay to $10^{-4}$ and $10^{-5}$, respectively. Regarding the setting of quantiles, we refer to EfficientAD and keep it consistent. For the logic anomaly detection module, the size of the feature extraction window of the MSOA module is set to three and five, respectively. At the same time, we refer to the normalization strategy of PUAD to integrate the unpicturable and picturable anomaly scores. A detailed description of the normalization approach can be found in Algorithm 1 and the study by Sugawara et al. [24]. In addition, for the hyperparameter α and the number of MSOA blocks, we conduct detailed experiments in Section 4.4 to determine the optimal default values.

### 4.3. Comparison with Other Methods

First, we conduct tests on MVTec LOCO to evaluate the logical anomaly detection capabilities of different algorithms. We compare the proposed method with several SOTA methods for structural anomaly detection, including SimpleNet [31], DRAEM [29], and PatchCore [22]. We also compare it with a number of SOTA methods for logical anomaly detection, including GCAD [10], ComAD [40], SINBAD [37], SLSG [41], LogicAL [42], and PUAD [24]. Additionally, our approach is compared with the baseline EfficientAD. The EfficientAD-M in the table denotes the medium version of EfficientAD. We remove the penalty constraint when retraining EfficientAD and PUAD.

Table 1 shows the AU-ROC results for the comparison test of different algorithms on MVTec LOCO. Our proposed method achieved the highest average AU-ROC value among all compared methods, while showing optimal detection levels for four of the five product categories. Methods such as DRAEM and PatchCore focus more on the local structural anomalies of the image and ignore the global relationships between the features in the image; thus, they score lower on MVTec LOCO. The GCAD, ComAD, and SINBAD methods focus more on logical anomalies, taking into account the global dependencies between image features or components, resulting in varying degrees of improvement. SLSG and LogicAL are designed to be optimized for both logical and structural anomaly detection, and the results on MVTec LOCO demonstrate that they achieve relatively good performance in logical anomaly detection. PUAD uses the former half of the student network for feature extraction, which has the advantage of not requiring additional computation. However, due to the insufficient ability of the PDN structure to extract global context features, our re-implemented PUAD achieves a suboptimal score. The proposed method minimizes the reconstruction differences between the student and the autoencoder with respect to the teacher’s model output. In addition, the well-designed feature extractor is more capable of extracting global features; thus, the proposed method exhibits the best AU-ROC results.

MVTec AD contains anomaly images for multiple product categories, and we test them on MVTec AD as well, with the goal of comprehensively evaluating the proposed algorithms’ ability to detect more products and multiple anomalies. The baseline for our comparison includes four SOTA methods focused on detecting structural anomalies, Dream, PatchCore, PaDiM, and SimpleNet, as well as EfficientAD, SLSG, LogicAL and PUAD, which are designed to focus on both structural and logical anomalies. Our training details remain consistent with those tested on MVTec LOCO.

Table 2 shows the AU-ROC results of the comparison test of different algorithms on MVTec AD. Compared with SLSG and LogicAL, which also take both logical and structural anomaly detection into account, LA-EAD not only achieves comparable performance in structural anomaly detection but also demonstrates superior performance in logical anomaly detection. It should be noted that LA-EAD is not optimized for structural anomalies, and the type of anomalies in the MVTec AD dataset is focused on structural anomalies of products, so the method does not improve the detection score of EfficientAD on this dataset. However, the drop in score for the ours is only 0.6%, which remains a competitive performance relative to the other compared methods. The table shows that SimpleNet achieves the highest average AU-ROC score among the compared methods. Nevertheless, as can be seen from Table 1, the capacities of SimpleNet to detect logical anomalies are poor. Overall, our proposed method achieves a good balance in the ability to detect logical and structural anomalies.

We notice that the average AU-ROC of the proposed method on MVTec AD is lower than that of EfficientAD, mainly due to a significant decrease in the Carpet class. For the Carpet category, the performance of both PUAD and the proposed method decreases, and the decline in precision of PUAD is more obvious. The Carpet class consists of texture-type images that contain some minute texture anomalies. Our analysis suggests that the logical anomaly detection module is insensitive to the local features of texture types, which will lead to a decline in performance on such images. However, compared with PUAD, the feature extractor we designed can relatively better extract local detail features. Therefore, for texture images like Carpet, the performance of the proposed method is significantly better than that of PUAD.

In addition to detection accuracy, inference latency is also an important consideration in industrial applications of the model. We compare the model with SOTA methods in terms of inference latency, image-level AU-ROC, and the number of parameters. The test results are shown in Table 3. In order to visually demonstrate the performance of the proposed method, we visualize the results in Figure 1. The results clearly show that our method achieves the best latency/accuracy trade-off.

### 4.4. Ablation Studies

We perform ablation experiments on MVTec LOCO for the components proposed in this paper to visually compare the change in anomaly detection capability.

The image-level AU-ROC scores on MVTec LOCO when applying our proposed methods are presented in Table 4. We first use EfficientAD as our baseline, and as in the previous experiments, we remove the ImageNet penalty constraint during training, corresponding to the first row of Table 4. “RDC (Proposed)” denotes the introduction of our proposed reconstruction difference constraint during the training phase. “LADM (PDN)” indicates that in the logical anomaly detection module, the student model is used to extract features just like in PUAD. Correspondingly, “LADM (proposed)” indicates the application of the proposed context feature extractor in the logical anomaly detection module.

According to Table 4, the introduction of RDC results in a 1.1% 1.8% improvement in AU-ROC, which verifies the effectiveness of the RDC in improving the logical anomaly detection. It should be noted that the official reported score of EfficientAD with the ImageNet penalty constraint preserved during training is 90.7 [23]. LA-EAD drops this constraint during the training phase, but the AU-ROC score remains better than EfficientAD, indicating that our method can achieve excellent detection capability without loading a large ImageNet dataset during training. This advantage makes our method easier to deploy and train across multiple devices in the factory. When comparing the effect of the feature extractor on the results, the results show that when applying the proposed global context feature extractor, our method achieves a better AU-ROC score compared to the way in PUAD. To summarize, both the reconstruction difference constraint and the global anomaly detection module proposed in this paper contribute significantly to the improvement of the detection accuracy.

**Figure 7:**
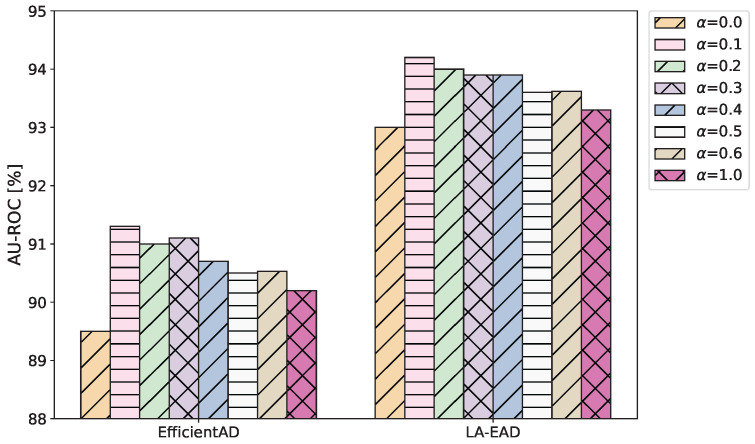
AU-ROC of EfficientAD and LA-EAD on MVTec LOCO under different settings of the α.

**Influence of hyperparameter α**: To evaluate the impact of the hyperparameter α of the RDC, this research conducts multiple experiments under different settings of α. Figure 7 reports the experimental results of EfficientAD and our method on MVTec LOCO. When α=0, the RDC has no effect, and the detection performance is the worst. After adopting the RDC, the detection performance of both EfficientAD and our method has improved to varying degrees. When α=0.1, the AU-ROC increases by 1.2%. Therefore, in this research, we set the default value of α to 0.1.

**Figure 8:**
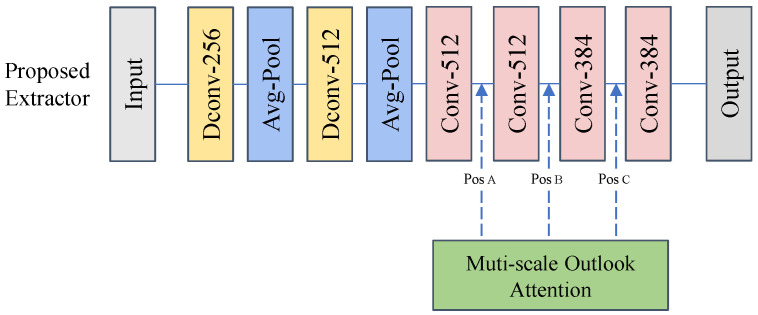
Schematic diagram of the MSOA blocks inserted into different positions of the feature extractor network.

**Influence of MSOA**: We investigate the influence of the position of the MSOA block in feature extractors. Figure 8 shows the three locations where we insert the MSOA block into the network. To maintain the low latency of the feature extractor, we insert the MSOA block behind the two global average pooling layers.

As presented in Table 5, the optimal position and quantities of MSOA blocks are illustrated. To explore how the number of MSOA blocks impacts logical anomaly detection performance, we conducted a series of experiments, and the results are detailed in Table 5. We introduce 0∼3 MSOA blocks respectively. “w/o MSOA” drops the MSOA block. “PosX + PosY” denotes different insertion positions and quantities of MSOA blocks. The results show that increasing the number of MSOA blocks has a limited effect on performance improvement. One possible reason is that a complex feature extractor is more prone to overfitting, thereby reducing the generalization ability of extracting anomalous features from images. Table 5 also shows the inference latency of the feature extractor under different settings. Due to the fact that the feature extractor with a single MSOA block achieves optimal performance, and adding MSOA blocks would result in increased latency, we ultimately chose the network architecture shown in Figure 6.

Furthermore, to further clarify the advantages of MSOA over other mainstream attention mechanisms, we compare it with SE attention [43], CBAM attention [44], and multi-head self-attention [45] (MHA) in terms of the number of parameters, FLOPS, and AUROC performance. The experimental results are shown in Table 6: compared with other attention mechanisms, MSOA brings a small increase in the number of parameters but exhibits significant advantages in performance, which indicates that it is more suitable for our framework to perform the logical anomaly detection task.

**Influence of the backbone architecture**: To investigate the sensitivity of our method to backbone network choices, we compared models with different backbones on MVTec LOCO and MVTec AD. The results in Table 7 show that LA-EAD variants (based on EfficientAD-S/M) outperform EfficientAD on MVTec LOCO, with scores of 93.8% versus 88.9% and 94.2% versus 89.5%. This confirms the framework’s robustness across different backbones. Although LA-EAD performs slightly worse on MVTec AD’s consistent gains on MVTec LOCO (for logical anomaly) indicate that our method can effectively adapt to different backbones.

### 4.5. Qualitative Analysis

**Figure 9:**
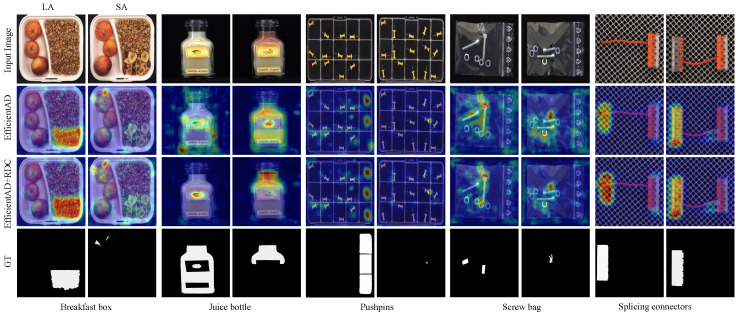
Score maps on the anomaly images of the MVTec LOCO dataset. We map the pixel values of the output maps to the 0-255 interval by min–max normalization and resize to 256 × 256 size.

We conduct a qualitative analysis of the proposed RDC on MVTec LOCO to visually evaluate the effectiveness of RDC in enhancing logical anomaly detection capabilities. As shown in Figure 9, the first row is input images, which contain all five product categories in the MVTec LOCO dataset. Each category is further divided into logical (left column) and structural anomalies (right column). The second row shows the anomaly score-map for EfficientAD without RDC in the training phase, while the third row indicates that RDC is used. The fourth row is the pixel-level labels of image anomalies. Visualization results show that the anomaly prediction score plots exhibit fewer false positives after using RDC, as shown by the comparison of the score-map for the Juice bottle class and the Screw bag class. Comparison of the prediction results between the Breakfast Box class and the Splicing Connectors class shows that the strength of the predicted response is improved by the introduction of RDC. Furthermore, the introduction of RDC has no negative impact on the predicted response to structural anomalies.

**Figure 10:**
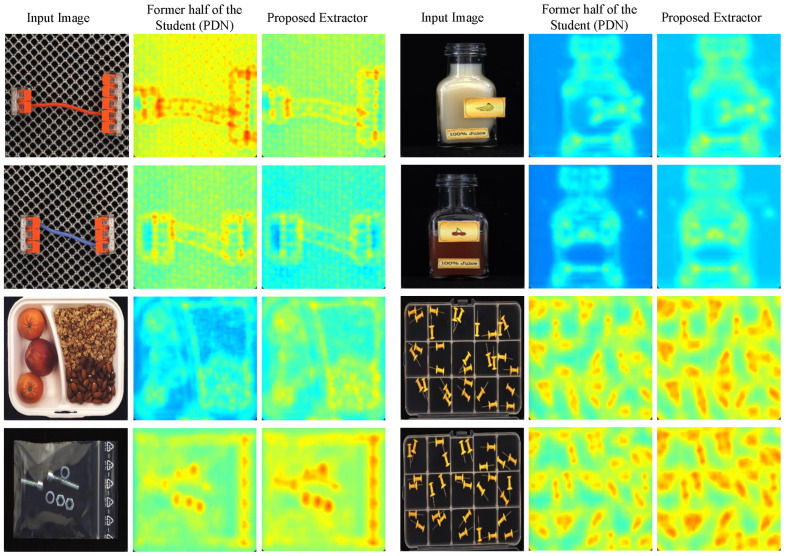
Comparison of feature extractors used in the global anomaly detection module.

In order to demonstrate the effectiveness of our proposed feature extractor, we compare it with the one described in PUAD that uses the former half of the student model as a feature extractor. We input an image from MVTec LOCO, and we obtain the output feature maps of two feature extractors separately, using the visualization method described in Figure 9. The student network is composed of traditional two-dimensional convolution blocks, which focus too much on local detail features in the image. Benefiting from the dilated convolution block and our designed multi-scale outlook attention module, our feature extractor pays more attention to significant object features and global contextual information in the image. As the output of the splicing connectors category in Figure 10, our feature extractor outputs a feature map that weakens the grid features of the background and extracts the connector features completely, indicating that the extractor focuses more on the prominent discriminative features. The results show that our extractor has a stronger response to the target features, which is beneficial for improving the discriminability of feature vectors in subsequent calculations of the Mahalanobis distance.

## 5. Discussion

According to the ablation experiment results in Table 4, in the MVTec LOCO benchmark test, the introduction of RDC increased the AU-ROC by 1.1-1.8%. This improvement is reflected in the visualization results (Figure 4 and Figure 9) as a reduction in the abnormal prediction response in the normal area, as shown in the Juice bottle category in Figure 9. The weight of the RDC loss in the overall loss is regulated by the hyperparameter α, and we conducted sensitivity experiments on this parameter. The results in Figure 7 show that when α>0 (the RDC loss is introduced), the overall performance of the model is significantly better than when α=0 (the RDC loss is not introduced). However, as α increases, the model’s performance exhibits a slight downward trend. We consider this may be because an excessively large α causes the student model to overly pursue consistency with the feature distribution of the autoencoder, thereby inadvertently bridging some feature differences that are actually indicative of anomalies. Based on these experimental results, we determined the optimal default value for α. Table 4 shows that the introduction of the logical anomaly detection module further significantly improves the AU-ROC. Compared with the original EfficientAD, the AU-ROC after the introduction of the logical anomaly detection module increases by 3.5%. This indicates that integrating feature-based methods in the architecture of EfficientAD can effectively enhance the ability of logical anomaly detection.

The visualization results of the feature maps extracted by different extractors (Figure 10) show that the proposed feature extractor pays more attention to the global discriminative features, thereby improving the inter-class discriminability of the feature vectors. The ablation experiment results in Table 5 further verify the effectiveness of the introduced MSOA module.

As shown in Table 3, compared with the original EfficientAD model, the proposed model achieves the highest detection accuracy, but with a slight increase in the number of parameters and inference latency, where the inference latency is 6.1ms, which still fully meets the industrial production requirements. Compared with other SOTA models, the proposed model has significant advantages in both inference latency and detection accuracy. This performance advantage stems from the fact that the proposed model inherits the lightweight structure of EfficientAD while not introducing excessive additional computational overhead.

However, there are still some limitations in our method. Firstly, the logical anomaly detection module takes image-level as the detection granularity. Therefore, our proposed method is unable to improve the pixel-level detection capability. Moreover, its performance drops when applied to texture-class images containing subtle anomalies. In future research, we will address the current limitation of being unable to achieve pixel-level logical anomaly localization. We plan to redesign the logical anomaly detection module to operate on image patches instead of entire images, aiming to generate spatial anomaly maps and thereby enable pixel-level logical detection. Additionally, for structural anomaly images with subtle defects, we will explore robust approaches to constructing anomaly sensitive features [46] and investigate adaptive mechanisms that dynamically weight structural and logical anomaly scores based on image characteristics, with the expectation of further improving the overall performance of the model.

## 6. Conclusions

In this paper, we propose the reconstruction difference constraint and the logical anomaly detection module to enhance the logical anomaly detection capability of EfficientAD. The reconstruction difference constraint is simply integrated during the training phase, which mitigates potential false-positive issues. We further integrate the feature-based approach to enhance the performance of logical anomaly detection and design a logical anomaly detection module for this purpose. To make full use of the global context information of the image, we introduce an additional context feature extractor and integrate it into our logical anomaly detection module. Extensive experiments show that our method achieves competitive performance, which proves that the method combining the reconstruction-based and feature-based methods has a good application prospect.

## Figures and Tables

**Table 1:** Anomaly detection AU-ROC [%] scores for different anomaly detection methods on MVTec LOCO. *: method designed for logical anomaly detection. Bold indicates optimal results.

Method	BreakfastBox	Juice Bottle	Pushpins	Screw Bag	SConnectors	Average
SimpleNet (CVPR 2023)	-	-	-	-	-	77.6
DRAEM (ICCV 2021)	80.3	94.3	68.6	70.6	85.3	79.8
PatchCore (CVPR 2022)	77.5	96.2	75.8	74.9	83.6	81.6
EfficientAD-M (WACV 2024)	83.9	97.7	96.3	72.6	96.9	89.5
GCAD (IJCV 2022) *	83.9	99.5	86.2	63.3	84.0	83.4
ComAD (AEI 2023) *	86.4	96.6	93.4	80.2	94.1	90.1
SINBAD (arXiv 2023) *	**91.8**	94.4	83.9	86.8	84.5	88.3
SLSG (PR 2024) *	88.9	99.1	95.5	79.4	88.5	90.3
LogicAL (CVPR 2024) *	85.4	98.5	87.4	82.0	89.0	88.5
PUAD (ICIP 2024) *	86.6	99.8	96.0	81.7	96.8	92.2
LA-EAD (Ours)	88.4	**99.9**	**98.8**	**86.9**	**97.1**	**94.2**

**Table 2:** Anomaly detection AU-ROC [%] scores for different anomaly detection methods on MVTec AD. *: method designed for logical anomaly detection. Bold indicates optimal results.

Category	PaDiM (ICPR 2021)	DRAEM (ICCV 2021)	PatchCore (CVPR 2022)	SimpleNet (CVPR 2023)	EfficientAD-M (WACV 2024)	SLSG (PR 2024) *	LogicAL (CVPR 2024) *	PUAD (ICIP 2024) *	LA-EAD (Ours)
Bottle	99.1	99.2	**100**	**100**	**100**	99.4	**100**	**100**	99.9
Cable	97.1	91.8	99.5	**99.9**	98.3	98.3	99.0	98.0	98.9
Capsule	87.5	**98.5**	98.1	97.7	98.0	95.5	95.5	97.8	97.1
Carpet	**99.8**	97.0	98.7	99.7	99.0	99.0	98.5	68.1	92.0
Grid	96.7	99.9	98.2	99.7	**100**	**100**	99.4	99.9	99.7
Hazelnut	99.4	**100**	**100**	**100**	99.9	99.5	98.5	99.8	99.9
Leather	**100**	**100**	**100**	**100**	**100**	**100**	98.1	99.9	**100**
Metal_nut	96.2	98.7	**100**	**100**	98.4	**100**	99.4	97.0	98.5
Pill	90.1	98.9	96.6	99	98.3	**99.2**	97.5	97.6	98.0
Screw	97.5	93.9	98.1	98.2	98.0	89.1	**99.3**	80.0	97.3
Tile	98.1	99.6	98.7	99.8	99.2	**100**	**100**	98.5	98.7
Toothbrush	**100**	**100**	**100**	99.7	**100**	**100**	**100**	**100**	**100**
Transistor	94.4	93.1	**100**	**100**	**100**	97.3	98.2	99.8	99.9
Wood	99.2	99.1	99.2	**100**	99.0	99.6	99.0	98.2	99.0
Zipper	98.6	**100**	99.4	99.9	97.0	**100**	**100**	97.1	97.5
Average	96.9	98.0	99.1	**99.6**	99.0	98.5	98.8	95.5	98.4

**Table 3:** Comparison of AU-ROC, latency, and number of parameters among different models on MVTec LOCO. Bold indicates optimal results.

Method	Parameters [$\times 10^{6}$]	Latency [ms]	AU-ROC [%]
GCAD	65	11.0	83.4
PatchCore	86	32.0	81.6
SimpleNet	73	12.0	77.6
EfficientAD	**21**	**4.5**	89.5
PUAD	**21**	4.7	92.2
Proposed	33	6.1	**94.2**

**Table 4:** Ablation experiments on the effect of the proposed components on performance. Bold indicates optimal results.

RDC (Proposed)	LADM (PDN)	LADM (Proposed)	AU-ROC [%]
-	-	-	89.5
-	-	-	90.7 (with ImageNet penalty)
-	🗸	-	92.2
-	-	🗸	93.0
🗸	-	-	91.3
🗸	🗸	-	93.3
🗸	-	🗸	**94.2**

**Table 5:** The impact of different positions and quantities of MSOA modules on performance. Bold indicates optimal results.

MSOA Settings	Latency [ms]	AU-ROC [%]
w/o MSOA	**0.7**	93.5
PosA	1.4	**94.2**
PosB	1.4	94.0
PosC	1.4	93.9
PosA + PosB	2.2	94.0
PosA + PosB + PosC	30	**94.2**

**Table 6:** Comparison of feature extractors integrated with different attention mechanisms in terms of number of parameters, FLOPs, and AUROC on MVTec LOCO. Bold indicates optimal results.

Extractor	Number of Parameters [$\times 10^{6}$]	FLOPs [$\times 10^{9}$]	AUROC [%]
Extractor-SE	**8.1**	**52.0**	92.9
Extractor-CBAM	**8.1**	**52.0**	92.8
Extractor-MHA	9.1	55.9	93.1
Extractor-MSOA (Ours)	12.5	68.1	**94.2**

**Table 7:** The impact of model’s backbone architecture on AUROC performance. Bold indicates optimal results.

Model	MVTec LOCO	MVTec AD
EfficientAD-S (based on PDN-S)	88.9	98.6
EfficientAD-M (based on PDN-M)	89.5	**99.0**
LA-EAD-S (based on EfficientAD-S)	93.8	98.1
LA-EAD-M (based on EfficientAD-M)	**94.2**	98.4

## Data Availability

The data presented in this study are available on request from the corresponding author.
